# “All emigrants are up to the physical, mental, and moral standards required”: A tale of two child rescue schemes

**DOI:** 10.1002/jhbs.22188

**Published:** 2022-02-08

**Authors:** Wendy Sims‐Schouten, Paul Weindling

**Affiliations:** ^1^ UCL London UK; ^2^ Oxford Brookes University Oxford UK

**Keywords:** British Home Child Scheme, child welfare, discrimination, eugenics, Kindertransport

## Abstract

The current paper critically assesses and reflects on the ideals and realities of two major (British) child migration schemes, namely the British Home Child scheme (1869–1930) and the Kindertransport scheme (1938–1940), to add to current understandings of their place within wider international histories of child migration, moral reforms, eugenics, settlement, and identity. Specifically, we focus on constructions of “mentally and physically deficient” children/young people, informed by eugenic viewpoints and biological determinism, and how this guided inclusion and exclusion decisions in both schemes. Both schemes made judgements regarding which children should be included/excluded in the schemes or returned to their country of origin (as was the case with children in the Canadian child migration scheme) fueled by a type of eugenics oriented to transplanting strong physical and psychologically resilient specimens. By viewing the realities of the child migration schemes, including the varied experiences and narratives in relation to child migrants, in light of eugenicist narratives of difference, pathology, victimhood, and contamination, we shed a light on uneven practices, formations of power, and expectations of the times.

## 1 INTRODUCTION

The history of British child migration schemes is long and fraught with scandals, contradictory motives, mixed reception, and political pragmatism (Sims‐Schouten, 2021; Constantine, 2013). By the late 19th century, children became a commodity who could be plucked from one location to another and readily transplanted. The current paper is centered around two child migration schemes, namely the British Home Child scheme (BHS, 1869–1930) and the Kindertransport scheme (1938–1940), and the controversies surrounding constructions and perceptions as to which children could and could not be rescued, inherent in each of the schemes (Sims‐Schouten et al., 2019; Craig‐Norton, 2014; Lynch, 2016). Specifically, we focus on constructions of “mentally and physically deficient” children/young people, informed by eugenic viewpoints and biological determinism, and how these guided inclusion and exclusion decisions in both schemes (Sims‐Schouten, 2021; Buss, 1976; Partridge, 1912; Stewart, 2009).

Between 1869 and 1930, over 100,000 juvenile migrants were sent to Canada as part of the British Home Child emigration movement, ostensibly centered around providing pauper children with a better chance for a healthy, moral life in rural Canada (Constantine, 2002; Jenkins, 2000; Parr, 1982). In a somewhat later time period (from December 1938 to August 1939), as part of the Kindertransport rescue scheme for children who were Jewish or had a Jewish background, a total of an estimated 10,000 unaccompanied children and young people (a notional number set by the British Government) arrived in the United Kingdom from Nazi Germany, with 2315 children from Vienna. This was followed by one further transport on May 14, 1940 to escape the advancing German invaders, organized by Geertruida Wijsmuller–Meijer, who had organized the first transport of 400 children from Vienna on 10 December 1938 (Craig‐Norton, 2014; Harris & Oppenheimer, 2001).

History is often used (and abused) in contemporary debates around child migration and child refugees, reinforcing Britain's image as “protector of vulnerable children” (Constantine, 2013). Yet, what the above schemes have in common is that judgements regarding which children should be included/excluded in the schemes or returned to their country of origin (as was the case with children in the Canadian child migration scheme) were fueled by eugenics and discriminatory selection procedures (Sims‐Schouten et al., 2019; Sims‐Schouten, 2020, 2021; Lynch, 2014). As an example, child emigration correspondence/archives in relation to the Canadian child migration scheme held at the Library and Archives Ottawa make reference to a boy who “is said to be deficient and the Canadian authorities wish to send him back.”^1^ Similarly, communication with the Home Office about the Kindertransport in 1939, specifies that this will only involve “children who are 100% mentally and physically fit.”^2^

Presented as a moral program to “rescue” children facing poverty or danger, the above highlights how the BHS simultaneously adopted discriminatory selection procedures, excluding “mentally inferior” and/or “physically impaired” children (Sims‐Schouten, 2021; Sohasky, 2015). Decisions about which children could be included in the BHSs were largely made by the sending homes, sometimes without parental knowledge, let alone consent (Coldrey, 1999; Constantine, 1991, 2002). Relevant decisions were recorded in “Chairman's Reports,” which referred to children/young people who were passed for emigration in terms of their “good nature,” character, intellect and physical capability. At the same time, children and young people who were “deferred” or “rejected” were often described in terms of being mentally and/or physically immature or deficient (Sims‐Schouten, 2021). Fueled by beliefs in biological determinism and eugenics, here the aim was to exclude children and young people with undesirable traits and characteristics, shaped by heredity (Buss, 1976; Partridge, 1912; Stewart, 2009). The goal was to distinguish healthy, “innocent” and “capable” children with hope for a future, from children for whom that innocence was complicated; the latter were perceived as in need of being managed and controlled, and excluded from the child migration schemes (Barham, 1999).

Similarly, in relation to the Kindertransport scheme, eugenic criteria of selection were used as part of the decision‐making process regarding which children could and could not be included in the scheme. Using the precedent of medical certification of fitness to migrate to Palestine, medical and psychological controls operated during the Kindertransport, something long overlooked (Weindling, 2020; Dashorst et al., 2019; Davidovitch & Shvarts, 2005). The reason for this lacuna is that knowledge of the Kindertransport studies has until recently largely been based on published memoirs of the “Kinder” themselves and oral histories. The administrative procedures used to select suitable children, have been overlooked (Craig‐Norton, 2014). There are documents demonstrating the need for parents to certify that the child was not suffering from a mental illness, infectious disease, nor was a bedwetter (Weindling, 2020). Moreover, driven by demographic considerations, the British authorities treated the Jewish child refugees as temporary immigrants and were reluctant to “replenish that good white stock with Jewish racial material” (Grenville, 2012, p. 4). It should be noted here that the notion of “white stock” was also central in decisions made in relation to the BHSs across the globe (Australia, New Zealand, South Africa, as well as Canada) where “good white stock” was requested to build the empire (Paul, 1995; Von Benzon, 2021). Both embody biological determinist viewpoints and racist eugenics, with the latter also reflecting white settler colonial projects (Kelly et al., 2021). Displaced children were frequently positioned within a lower social class/hierarchy and stigmatized as less important than other children, but still preferable to their indigenous counterparts (Ala, 2018; Hopkins & Hill, 2010).

There is a need to engage in a critical reflection of the ideals and rationales of historic child emigration schemes, which were presented as moral programs to rescue children facing poverty or danger, but which were in reality fueled by discriminatory selection procedures framed within stereotypes of mental inferiority and deficiency (Sims‐Schouten, 2021; Sohasky, 2015). This also means engaging with challenges in relation to conflicting research findings and experiences, which need to be placed in context. For example, although cases of abuse and neglect are widely reported in relation to the Canadian child migration scheme (see Constantine, 1991; Independent Inquiry Child Sexual Abuse [IICSA], 2018; Lynch, 2016), there are also writings that stress the altruistic motives of the agencies that sent children abroad. An example of the latter is the book on the “Middlemore Experience” by Roberts‐Pichette (2016), which constructs the Canadian child migration scheme in terms of an initiative that helped vulnerable children thrive.

The same can be seen with regard to Kindertransport. Although by some (including many of the “Kinder” themselves) this is hailed as an altruistic initiative, others show a more sinister story of bad treatment and racism (Weindling, 2020; Guske, 2009; Harris & Oppenheimer, 2001; Kushner, 2012). For the Kindertransport, each child required a guarantee of £50 (to be held by the Board of Deputies of British Jews to ensure that any refugee from Nazi persecution—the guarantees were also for adults—would not be a burden on public welfare) and there was a frantic search for host families. Here, it should be noted that the Board of Deputies, the largest and second oldest Jewish communal organization in the United Kingdom, established in 1760, held the guarantee rather than the UK government (Gewirtz, 1991; Langham, 2010). Although many of the receiving families provided good standards of care, some expected children to work as unpaid domestics and there were cases of sexual abuse (Fast, 2011). In February 1939, the upper age limit for the Kindertransport was twice reduced from having to be under 18 years and then under 17 years, so as to steer children into agriculture and nurse training, both areas of labor shortage. Likewise, the majority of the children sent to the receiving homes in Canada were eventually sourced out to work—boys on the farms and girls in domestic service (Lynch, 2016; Parr, 1982).

This study draws on archival data (correspondence, case files, emigration paperwork, reports, and magazines) associated with children and young people sent to Canada by the Waifs and Strays Society and the Fegan Homes, and children/young people who were sent to the United Kingdom via the Kindertransport scheme from Vienna, where the records of the administering child welfare department of the Jewish community (Israelitisches Kultusgemeinde) have survived extensively. Here we focus specifically on language around mental, physical, and moral deficiency. The data (Fegan Homes and Waifs and Strays Society) linked to the BHS was accessed at two sites, namely at the Children's Society archives in London and Library and Archives Canada (LAC) in Ottawa. A total of 100 children's case files associated with the Waifs and Strays Society (consisting of correspondence from custodians, educators, medical officers, church reverends, practitioners linked to asylums and industrial schools, as well as parents and children) were accessed at the Children's Society Archives in London. In addition to this, 42 microfilm reels (consisting of roughly 1500 images each), comprising minute books, emigration papers, and correspondence between receiving and sending homes, associated with both the Fegan Homes and Waifs and Strays Society, were accessed at the LAC. Data linked to the Kindertransport scheme was accessed via the Central Archives for the History of the Jewish People in Jerusalem, the Jewish community archive in Vienna, as well as the Wiener Library in London. The Vienna Jewish child welfare department assessed each child in terms of educational attainment and the level of parental care, to determine urgency of the particular case. The 12 cartons (each carton containing often 20 folders of ca. 200 original records and child photos) are in the Central Archives for the History of the Jewish People in Jerusalem and there are microfilms of the collection at the Jewish community archive in Vienna.^3^ Data collected at the Wiener Library (Collection 1368) consists of Reunion of Kindertransport documents (1987–2002) providing insight into the function and character of a child survivor group, and more broadly reflects an aspect of Holocaust remembrance 50 years on.

The current paper puts issues of child welfare and migration in a wider historical, political, and psychological context. By critically reassessing and reflecting on the ideals and realities of two major (British) child migration schemes, we aim to add to current understandings of their place within wider international histories of child migration, moral reforms, eugenics, settlement, and identity. This includes a critical reflection of the varied experiences and narratives in relation to child migrants, as well as the ideals and rationales of emigration schemes, which were presented as moral programs to rescue children facing poverty or danger of traumatizing, racially degrading and ultimately genocidal persecution. Within this, there is a need to open up eugenicist narratives of pathology, victimhood, and contamination, including uneven formations of power and expectations that informed the processes involved in the decision‐making and the experiences of individual children therein (Kelly et al., 2021; Lynch, 2016; Moss et al., 2020). In the next sections, we introduce the two schemes, as well as discussing commonalities and differences between the schemes. After this, we discuss the notion of “mentally and physically deficient” children/young people, followed by examples in relation to each of the two schemes.

## 2 THE TWO CHILD RESCUE SCHEMES

The current section provides an overview of the Canadian BHS and Kindertransport, both in terms of commonalities and differences. Presented as “child rescue schemes,” the realities of the children's experiences often diverged from the idealized ways in which welfare organizations presented life in the “promised land,” including the dependence on child labor to cover the costs of emigration (Craig‐Norton, 2014; McDonald, 2018; Soares, 2016). The latter was true for both the BHS and Kindertransport scheme. For example, although young people sent to Canada by the many philanthropic institutions in Britain were “placed in homes in the dominion; the boys as farm workers and the girls as domestic helpers,”^4^ testimonials from former Kindertransport children also highlight involvement in child labor:

*I left Berlin on abt. July 19, 1939, as a child of nearly 15 years old. We were a large group of all kinds of children all ages. My first stop was Great Engham Farm near Kent, where we were transferred to North Wales. From there we were separated and put in different places in order to work for Agriculture Committee. I changed a lot of places till in the end I landed in London in a place called Cazenove Road. All those nine years we worked on the land or in the House as DOMESTIC*.^5^

Indeed, as the upper age limit was lowered twice in February 1939 from 18 to 16 years, it became an official policy for the older boys to be physically assessed for an agricultural training scheme with a view for ultimate resettlement in Palestine (as a British Mandate) and for girls to obtain posts in a parallel domestic or nurse training schemes (Weindling, 2020; Craig‐Norton, 2014).

So far, we have highlighted the commonalities between the two schemes as reasons for our comparison of the schemes, namely the ways in which both schemes are constructed as British child rescue schemes, reinforcing Britain's image as “protector of vulnerable children” on the one hand and the discriminatory selection procedures and link with child labor inherent in each scheme on the other (Sims‐Schouten, 2021; Weindling, 2020; Constantine, 2013). Nevertheless, it would also be good to point out that both schemes differ in a number of ways. First, although both schemes were constructed as child rescue schemes, they had different objectives, the role of the BHS was to “rescue” children from impoverished and “unfit” families, whereas the Kindertransport was aimed at rescuing children from the Nazi regime. Second, the difference in numbers should be noted, with over 100,000 children/young people being sent to Canada as part of the BHS and merely 10,000 children/young people included in the Kindertransport scheme (although both schemes were marked by huge numbers of rejections). Third, the BHC to Canada needs to be viewed against the backdrop of Canada as a settler‐colonial nation with its own history of eugenics involving indigenous people, especially children, which was very different from the Kindertransport scheme (see also Neeganagwedgin, 2012). Finally, although Britain's 2010 apology to British Home Children suggests that the troubling “reality” of that program is in fact in view, this is very different for the Kindertransport (Craig‐Norton, 2014; IICSA, 2018). Below we provide an overview of the two child “rescue” schemes—the BHS and the Kindertransport scheme.

### 2.1 BHS

Motivated by social and economic forces, churches and philanthropic organizations sent orphaned, abandoned, and pauper children to Canada between 1869 and 1930 (Sims‐Schouten, 2020; Constantine, 2002; Jenkins, 2000). Many believed that these children would have a better chance for a healthy, moral life in rural Canada, where families welcomed them as a source of cheap farm labor and domestic help (Lynch, 2016; Parr, 1982). At the same time, there was an element of hostility from trade unionists and child care providers in Canada, who, influenced by eugenics, reproached child migrants as degenerate “slum kids” (Constantine, 1991; Partridge, 1912; Stewart, 2009). Thus, the motivation of the British sending Homes, to “rescue” poor, pauper children and young people, and provide them with a “better” chance in Canada was met with contempt and disapproval of the potentially deficient (mentally and physically) nature of the emigrants (Sims‐Schouten, 2020). To justify the UK child migration schemes to Canada, especially in light of this public opposition to these schemes in Canada itself, the British Philanthropic institutions in charge of the schemes adopted a narrative of supplying Canada with “honest” and “industrious” youth (Barham, 1999). In addition to this, there was an attempt to silence critics by stressing that certain children were capable of redemption (Lynch, 2014).

Thus, the BHSs revolved around conflicting narratives of “honesty,” “capability,” and “ability” on the one hand and “deficiency,” “dishonesty,” and “incapability” on the other. The latter comprised anything from being “mentally deficient” through to “bedwetting,” and physical ailments and disabilities, which were perceived as undesirable symptoms of social and physical degeneracy (Sims‐Schouten, 2020; Sims‐Schouten et al., 2019; Barham, 1999). In this case, this meant exclusion from the child migration schemes. It should be noted however that the moral framings and eugenics that influenced decisions regarding which children and young people should be included/excluded from the schemes coexisted alongside paternalistic and economic judgements, that is, children as sources of cheap farm labor and domestic help, in complex and often contradictory ways (Lynch, 2016; Parr, 1982).

Moreover, it has been well‐documented that children and young people who were included in the scheme were often poorly treated and abused following arrival in Canada (IICSA, 2018; Lynch, 2014), whereas some others experienced a better life there than if they had remained in the urban slums of England (Roberts‐Pichette, 2016). The child migration schemes were run by philanthropic agencies—two such voluntary institutions, which this paper will focus on, were the Fegan Homes and the Waifs and Strays Society; the first was responsible for sending 3200 boys to Canada, between 1884 and 1915, and the latter for sending ~3500 children (both boys and girls) to Canada between 1883 and 1937 (Global Heritage Press, 2013; Kohli, 2003). The reception of children in Canada by child welfare societies was couched in phrases professing the needs of the child, while at the same time negotiating the needs of the prospective foster home. In essence, British boys and girls were sent unaccompanied to Canada, indentured to work as agricultural laborers and domestic servants: “Doption, sir, is when folks gets [sic] a girl to work without wages” (Parr, 1980, p. 82). The Canadian child migration schemes came to an end in 1930 (although the scheme continued in other parts of the world, such as Australia, till the 1970s), when Canada ruled that no child under 14 years would be admitted to the country, unless accompanied by parents. This legislation was the result of changes in Canadian economic conditions and dissatisfaction with the labor provided by the children/young people, as well as increased criticism from child welfare societies against the “philanthropic abduction” of children/young people (Constantine, 2002; Hammerton, 2017).

### 2.2 Kindertransport

Between 1938 and 1940, the Kindertransport movement, a government‐facilitated yet privately funded scheme, brought 10,000 unaccompanied, mostly Jewish, children from Germany, Austria, Czechoslovakia, and Poland to the United Kingdom (Craig‐Norton, 2014). The movement has been celebrated as a humanitarian act of rescue by the British government and people on the one hand, and as motivated by political pragmatism rather than humanitarian compassion on the other, ignoring the spiritual and emotional needs of the children (Kushner, 2012; Skelton, 1944). Similar to the BHS, the Kindertransport scheme was permeated by paternalistic, classist, and discriminatory attitudes. For example, the expectation of domestic help from teenage girls reflected societal attitudes about refugees, status, and gender, and labor was treated as a fair recompense for rescue—the natural lot of charity cases who had been rescued by the good graces of British people (Craig‐Norton, 2014; McDonald, 2018). Moreover, domestic work and nursing were effectively the only routes to a British visa for Jewish young women and twice as many Jewish refugees arrived in Britain via this route than through the Kindertransport. As such, it follows that the presence of a large number of young Jewish maids in Britain may have blurred the distinctions between this group of refugees and older Kinder girls and has resulted in some foster parents regarding their Kinder as workers rather than as family (Craig‐Norton, 2014).

As with the BHS, there is evidence that the Jewish migrant child was often seen as synonymous with the “less worthy,” and children perceived as in poor health or mentally deficient were excluded (Weindling, 2020; Kushner & Knox, 2012; McDonald, 2018). Kushner and Knox (2012) argue that the image of the Kindertransport that has survived in British public memory (i.e., the notion that the Jewish child refugees were welcomed) is selective and flawed, and instead the Kindertransport was marked by marginalization, refused entry, and exclusion (see also McDonald, 2018). Indeed, as with the children included in the BHS, it would be wrong to imply that all Kindertransport children experienced growing empowerment and successfully negotiated the transition from dependence to autonomy. Moreover, a specific subgroup of children, namely children and young people with “mental deficiencies” referred to as “mental defectives” were consistently excluded from both the BHS and Kindertransport schemes.

## 3 EXCLUDING “MENTALLY AND PHYSICALLY DEFICIENT” CHILDREN AND YOUNG PEOPLE

The period of the late nineteenth to early 20th century was marked by significant reforms in ideas and practices pertaining to children (Sims‐Schouten, 2020; Hendrick, 1997). A central part of this was the focus on “normality” and “abnormality,” leading to three distinct forms of “normal” childhoods, namely normal as healthy, as average, and as acceptable. Using “normal” as the baseline, “abnormality” was initially associated with physical traits and deficiencies, but became increasingly synonymous with perceived deficits in mental capacity, personality, and conduct (Sims‐Schouten, 2021; Wright, 2001). Perceptions around physical and mental abilities and deficiencies were highly pertinent in decisions regarding which children should be included or excluded from various child rescue and migration schemes—in this case, the BHS and Kindertransport (Fong et al., 2018; Jones et al., 2018; Sohasky, 2015). The focus here was largely on a reductionist or isolated notion of the individual, who was blamed for being “mental deficient or defective” and/or “physically impaired” and “not strong enough,” and therefore not entitled to support or inclusion in the relevant migration scheme (Dagnan, 2007; Toms, 2012). This was underpinned by widespread beliefs that child development was irretrievably shaped by heredity, and that “defective” members of the population cause a general deterioration of the racial stock, unless kept strictly controlled and segregated (Weindling, 2020; Harris, 1993). This included not only those who had a mental deficiency or physical disability, but also those who were believed to present a “moral threat” to the society.

Despite the growing understanding and acknowledgement of the role of multiple factors in childhood (psychiatric) disorders in the early 1900s, such as the role of the family and early experiences (e.g., see the work of Freud, 1989; and Fairbairn, 1952), the main focus was still on heredity (Fuller, 1985; Raines, 2014). G. Stanley Hall, an American psychologist with a specific interest in child development and eugenics, made studying children a priority in science, and his 1904 book, *Adolescence*, which drew attention to the role of heredity and environment in moral development and psychopathology in childhood, was widely read across the Western world, including Great Britain (Sims‐Schouten, 2020; Hall, 1904; Partridge, 1912; Stewart, 2009). The eugenics movement, rooted in the biological determinist ideas of Sir Francis Galton, gained momentum during the early 1900s (Sims‐Schouten, 2021; Buss, 1976). The child migration schemes were heavily influenced by the assumption that children's traits and tendencies (moral, physical, and mental) were irretrievably shaped by heredity (Partridge, 1912; Stewart, 2009). By only including children who were mentally and physically able, and “good white stock” (in the case of the BHSs), the child migration schemes distinguished “innocent” children with hope for a future, from children for whom that innocence was complicated. Children and young people who we would recognize today as having learning disabilities were “graded,” with the “idiots” (the profoundly disabled) at the bottom, followed by “imbeciles” (the “medium grade”). In addition to this, there were two further types, namely the “feebleminded,” those who were mildly disabled yet able to contribute to their own support, and the so‐called “moral defectives,” those who “cannot distinguish right from wrong and represent a grave danger to the community” (Borsay, 2005; Walmsley, 1999). The Mental Deficiency Act of 1913 and the Act of Parliament of the UK, invented a whole new category of disabled person—the “moral imbecile.” The categories of “moral imbecile” and “moral defective” survived until 1959. Here, disability had nothing to do with the shape of a person's body or their intellectual capacity, instead it was about how a person thought and behaved, and the potential threat this caused to the population integrity (influenced by eugenics and related beliefs) (Chettiar, 2012; Galton, 1865; Singh & Tuomainen, 2015; Slack & Webber, 2008; Thomson, 1998). Thus, disability was constructed as unwanted, pathological, and something to eliminate. Not only that, this narrative of pathology extended onto other groups, namely the indigenous, racialized, and poor people (Kelly et al., 2021).

For example, in the case of the BHS, the desire to have mentally and physically able White British children/young people was aligned with colonial motivations to expand the “white stock” at the cost of the First Nations people. At the same time, it was racial and anti‐Semitic motivations that drove the desire to limit the number of Kindertransport children/young people to enter the United Kingdom, with the ones who were allowed in having to comply with strict rules of entry (i.e., only the mentally and physically fit) (Craig‐Norton, 2014). It should also be noted that although currently viewed by most as reflecting faulty scientific thinking, eugenic thinking itself consisted of many layers and influences, with British eugenics predominantly centered around socioeconomic status and US eugenics on race‐based theories of physical traits; Canadian eugenics was derived from both British and US eugenics (Kelly et al., 2021; McLaren, 2015).

Both schemes were marked by the type of exclusion practices discussed above and in both schemes the mental and physical “fitness” of the child needed to be evidenced, either by the sending Homes and philanthropic institutions (in the case of the BHS) or the parents, guardians, or Home in the case of children deposited in orphanages (in the case of the Kindertransport scheme). In addition to serving biological determinist and eugenic narratives, this focus on physically and mentally fit children served an additional purpose as well, namely that of child labor. In the case of the Kindertransport, although an ability work was not a prerequisite, some receiving households expected children to work as unpaid domestics (Fast, 2011). In the case of the BHS, it was clear that an ability to work was in fact a prerequisite for the one‐way passage to Canada. Parr (1980, p. 82) highlights that this is also something that was openly admitted by participating officials:

*We are not so young and unsophisticated as to imagine that the farmers take our boys for love…. The primary object of the farmer in taking a boy is that his services be useful to him*.

The BHSs were run by philanthropic agencies, for example, the Barnardo Homes, Waifs and Strays Society, Fegan Homes, and Middlemore Homes, to name a few. Decisions about emigration to Canada were generally taken by the sending Homes in collaboration with founders of the specific Homes/Societies (Coldrey, 1999; Constantine, 1991, 2002). The current study focuses on the Fegan Homes and Waifs and Strays Society. Both the Fegan Homes and the Waifs and Strays Society maintained strict policies, on paper, regarding child emigration, requiring consent from parents/guardians, as well as input from sending and receiving Homes. Yet, in reality, children were often sent to Canada without parental consent and it is not possible to discern whether children without parents or a guardian were more likely to be proposed for emigration compared with other children (Bagnell, 2001; Parker, 2010). The fact that at times parental consent was not included in child migration decisions was justified by the need for protecting children from “immoral” parents or other family members, by dispatching them to new homes and new lives overseas (Coldrey, 1999; Constantine, 1991, 2002). In the case of the Kindertransport, there were children who had lost one or both parents due to illness, or had “stateless” parent/s deported often to Poland but had left a child/children in Germany, or had managed to obtain an emigration visa for themselves but had left children behind (although more often the parent/s were left behind) (Weindling, 2020). There were many pressures that fractured family units.

Decisions around which children/young people could and could not be included in the child migration schemes to Canada consisted of different levels of communication, from initial correspondence and reports/letters of sending homes flagging up the potential of emigration, through to “Chairman's reports” on “cases of emigration,” which centered around specific outcomes (e.g., “passed” or “deferred” for various reasons, including physical ailments/disabilities, “mental deficiencies,” “not strong enough” or “not to be trusted”) (Sims‐Schouten, 2021). Parental consent did not feature much here—instead the focus was predominantly on the child's mental and physical fitness, which also needs to be viewed in light of opposition to these schemes in Canada, and the condemnation of child migrants as degenerate “slum kids” (Constantine, 1991; Lynch, 2014). Thus, justifications for why children were sent to Canada needed to be supported by declarations of mental and physical fitness, and strict records were held here. For example, correspondence in relation to a girl who had been looked after by the Waifs and Strays Society since she was 4 years old in 1889 highlights how emigration to Canada was contemplated and dismissed due her being: *“*painfully slow and stupid, but I considered her mentally deficient” regardless of her nature and behavior: “She is said to be a good girl, willing to work and well‐conducted.” “This girl will not be suitable for emigration to Canada.”^6^ These records started while the child was still in the United Kingdom, and records and Chairman reports were also continued when the child arrived in Canada. In many cases, an unsatisfactory report meant that the child would be returned to England. For example, correspondence from a Waifs and Strays home in Canada refers to a *“*Bad report of Norah Cadd, aged 17” and that “efforts are being made to find her another home.”^7^ If this continued to be unsatisfactory, this could then lead to the young person being returned to England, regardless of time spent in Canada, as was the case with Victor: “Victor Thatcher, said to be deficient. Emigrated in 1903. From the case paper it would seem that we ought not have sent him.”^8^ This report is followed by the following in 1911: “A boy named Thatcher who has been in Canada nearly 8 years–emigrated from Lambeth Workhouse–is said to be deficient and the Canadian authorities wish to send him back–but he has been out in Canada over 7 years and will be 21 years old next June.”^9^

The Chairman's reports, which were so diligently kept, played an important role here, as it was here that the emigration decisions and reasons for these decisions were recorded, reflecting thorough checks of the kind that should prevent issues, such as Thatcher's case, from happening too often. Chairman's lists and reports usually consisted of brief references to the child/young person and migration decision. For example, although Henry J Collins aged 13 years and 3/4 was passed for emigration (no date), Albert E. Jones, aged 14 years old was not passed “owing to his requiring a special surgical boot,” and Thomas Patterson was not passed, because “cold could make eczema worse.”^10^ At the same time, both Matilda A. Kewton (aged 14 years old) and Alice Turvey (aged 14 and 3/4) were deferred “in hope that purloining will be overcome,” whereas Ethel A.J. Bailey (aged 17 years old) was deferred, because she was “not to be trusted.”^11^ Ernest A. Goodall, aged 13 and a half years was deferred due to incontinence and a number of other young males, all aged 13 and a half years, were deferred due to being “rather under age.”^12^ In addition to this, similar to Ethel A.J. Bailey, a number of young people were rejected and referred to as “not to be trusted” or “not strong enough.”^13^

The moral framing of supplying Canada with “respectable” youth, able and with good disposition and “not in any way mentally defective”^14^ was designed to silence potential critics and can be seen in correspondence and case files relating to the young people prior and after arrival in Canada (Sims‐Schouten, 2021; Lynch, 2014). For example, the Fegan Homes in Canada performed regular check ups and held meticulous records of the young boys' behaviors and progress, while in their care, and references were made to “Character from the Home–Grand, worth his weight in gold–of most respectable Christian family”^15^ as well as “A really fine boy in body mind and moral character–shy at first.”^16^ Moreover, prior experiences were also acknowledged here, such as in relation to a young German boy, sent to Canada via an orphanage in London:

*A boy of German parents who suffered considerably through being aliens during the war. Since his father's death Paul has been brought up in a German orphanage in London–formerly well maintained, but in late years the funds have fallen considerably. He has much improved on the farm. Has a pleasant disposition, and will develop satisfactorily*.^17^

All throughout the records, of both the Waifs and Strays and the Fegan Homes, it is made very clear that the policy of only sending out children and young people who were up to the required physical and mental standards was strictly adhered to:

*allemigrants are up to the physical, mental and moral standard required by the Canadian Government Authorities, and capable of earning their own living*.^18^

Moreover, representatives from the sending homes in the United Kingdom, including the founders (e.g., Mr. Rudolph who founded the Waifs and Strays Society) would travel to Canada a couple of times per year and report back on the state of affairs through a lengthy letter referred to as “Impressions.” An “Impressions” letter dating August 19, 1929, highlights some trouble and discontent in the receiving homes, especially when it comes to the mental and physical fitness of some of the young girls:

*if this is to continue greater care must be exercised in the selection of girls, and all candidates should be examined by our own doctor to see if they are mentally as well as physically fit. There is no doubt about it, girls have been sent out because they have had undesirable relatives. Also in filling up reports some matrons have not always been truthful, and a troublesome girl is pushed through. In the last party a girl who for years must have been a ‘bedwetter' was sent out*.^19^

It is clear that the philanthropic organizations and “child rescue” Homes had to engage with conflicting narratives, between their “duty” to rescue children and young people from their unsatisfactory families in England and the expectation to only send out mentally and physically fit children and young people to Canada (Sims‐Schouten, 2021). In line with eugenicist perceptions of the time, a narrative of pathology was used to define, frame, and contain the young people, both as victims and contaminants/burdens, morally warped by impairment (Kelly et al., 2021).

## 4 “WE CANNOT BRING MENTALLY RETARDED CHILDREN UNDER ANY CIRCUMSTANCES TO ENGLAND”: EXAMPLES FROM THE KINDERTRANSPORT SCHEME

Similar to the BHS, the Kindertransport scheme also required evidence that the children and young people included in the schemes were of good mental and physical health. For example, Lola Hahn‐Warburg, who administered the central coordinating committee, “Movement for the Care of Children from Germany,” later simply the Refugee Children's Movement (RCM) in London, collected data around the medical status of a child and his or her educational attainment with a copy of the school record (often the child's last as Jewish children were ejected from the school system)^20^. The Vienna Israelitische Kultusgemeinde: Jewish Community of Vienna (IKG) Children's Department social work reports were less critical than required by the RCM, seeking positive attributes in the hope of lifting the child out of a situation of domestic deprivation brought on by the Nazi authorities cutting pensions and all welfare benefits for Jews, as well as closing down Jewish businesses. The IKG was faced by a Jewish community under severe stress, economically because the Nazis had created mass unemployment among Vienna's Jews, terminating pensions and welfare entitlement (Heimann‐Jelinek et al., 2007; Rabinovici, 2011).

The medical certificates issued in Vienna took various formats. The simplest was a parent certifying that the child was not suffering from a mental illness, infectious disease, nor was a bedwetter. Some certificates were provided by the so‐called Heilbehandler (Treater of the Sick—as Jews were stripped of the title of Arzt or physician) Dr. Heinrich Moser—one Leo Topper received such a certificate—and by the Medical Adviser to the British Consulate. Another certificate came from the Jewish community hospital in Vienna; this stated, as early as September 5, 1938, that “a hip patient needed additional postoperative care.” Later, self‐certification by parents was deemed acceptable. Dagobert Klein was rejected on January 18, 1939, as unsuitable, because he was diabetic.^21^ Hahn‐Warburg's assistant, Grete Exiner, asked on March 16, 1939, whether Kurt and Karl Kramer were mentally healthy.^22^ Hahn‐Warburg wanted high achieving children. Certain types of children were sought after: Kitty Milch was commended on May 1, 1939, “an intelligent looking and not particularly Jewish young woman.”

Those deemed to have physical defects faced rejection by the RCM: the mother of a blind child, Jenta Feuer, aged 8 years, wrote to Dr. Löwenherz of the IKG on August 15, 1939, but he was flatly turned down by the RCM. Jenta was killed on August 15, 1942, at the extermination camp, Maly Trostinec near Minsk. This pattern of rejection by the RCM and later deportation to a holding ghetto or to immediate killing is repeated time and time again (Hecht et al., 2019). One child, Elfriede Rerucha, who exceptionally survived deportation to Riga, can be identified. Unless adolescents migrated illegally to Palestine, or their parents managed to extricate themselves either with a US affidavit or to the exceptionally visa‐free Shanghai, children were killed. For children with any form of disability, they could be sent to a killing institution, notably the Spiegelgrund “*special child care institution*,” where murdered children's brains were retained for research, or to a psychiatric institution where until mid‐1941 adolescents were killed in the T4 killing program, for those from Vienna at Schloss Hartheim in Lower Austria where carbon monoxide gassing was installed.^23^ The London Movement/RCM insisted repeatedly and unrelentingly that only physically and mentally healthy were allowed because of the assurances given to government officials in the United Kingdom. No educationally backward children, no blind or deaf, and no physical disabilities were accepted. Exiner wrote to Schwarz on January 2, 1939, how the Movement had promised the Home Office to take only 100% physically and mentally fit. Hahn‐Warburg also insisted that “We have given the Home Office an undertaking that the Movement will only bring children who are 100% mentally and physically fit. This applies also to guaranteed children.” Exiner wrote on March 10, 1939: “We cannot bring mentally retarded children under any circumstances to England, even when they attend a normal school.”^24^ Such views fitted in with the UK's “Mental deficiency Act” of 1913. It meant that there was a succession of declined and excluded children, including Hilda Loebl, aged 15 years, and her sister Rosi, aged 8 years, and Manfred Frisch, Isaac Habermann, Ernst Nadel, and Eduard Friedl, all described as “mentally backward.” Georg Lipschitz was pronounced “geistig minderwertig” (mentally inferior) and the RCM returned his papers in August 1939.^25^

Ernst Lerner, born August 24, 1932, was a half‐orphan and his mother was blind. He was reported by the Jewish Boys Orphanage to be “very well‐behaved and tranquil” (“sehr brav und ruhig”) but “very slightly mentally backward” (“jedoch in seiner geistigen Entwicklung etwas zurückgeblieben”). He was similarly rejected by the RCM. He was deported to the extermination camps of Majdanek or Sobibor on June 14, 1942.^26^ Gerda Kral was judged on August 22, 1939, to be “nicht ganz vollwertig” (“not quite fully fit”), although her speech incapacity derived from having contracted diphtheria. Exiner sent her papers back, because the RCM, as she stated again, could only accept 100% mentally normal children.^27^ Gerda was deported to Riga (a site of mass shootings) on February 6, 1942. Being classified as mentally defective was a death sentence: the Vienna IKG Children's Department had a greater sense of the peril facing Vienna's Jewish children then the RCM.

A more fortunate case was that of Hilde Goliath. Her guarantor (Mrs. Hough) contacted the British Consulate in Vienna on April 18, 1939, to bring Hilde over. Hilde's mother had a breakdown and committed suicide 2 days later. Hilde's father had been in a concentration camp since November 10, 1938 (and was eventually killed in custody). The Movement objected that Hilde's mother suffering from a “high degree of nervous illness” (“schwer nervenleidend”) excluded Hilde's coming to the United Kingdom. The IKG office in Vienna presented the case as one of “cold and starvation,” as well as persecution. When the RCM asked whether the child was mentally normal, the IKG responded that “the child is wholly normal” (“das Kind ist vollkommen normal”), based on her school record.^28^

A similar case was that of Herta Baumfeld (born April 26, 1926), whose father was in “protective custody” (often a euphemism for a concentration camp) and mother in a psychiatric institution. Herta was living with her infirm grandmother. The Vienna IKG office wrote on Herta's behalf on July 11, 1939.^29^ Herta was killed at Maly Trostinec on September 18, 1942. By way of contrast, there was a demand for healthy young children, who should preferably be orphans, for adoption in the United Kingdom. There was some porosity and some less able children made it to England, yet as with the BHS children, their experiences were not always straightforward. After release from the concentration camp of Sachsenhausen, Felix Reich brought over 10 deaf children from Berlin, including Ann and Horst Marschner, who were then schooled at the Residential Jewish School for the Deaf in Nightingale Road, Balham, London.^30^ Another rescued boy was Hans Albrecht: Hans Albrecht was born in Linz, Austria on April 27, 1931. He was rescued from the Nazis shortly before World War II by being brought to England on the Kindertransport in May 1939. As a child with learning difficulties and autism, Hans had a difficult experience, moving frequently between foster carers and with his mother for brief periods after her escape from the war.^31^

If children were “guaranteed” (having a family or organization prepared to deposit the £50 with the Board of Deputies of British Jews), and contrary to what the RCM alleged, they could be admitted to the United Kingdom. However, emigration was far harder for families with disabled children and there are cases when the disabled were left behind. In other situations, it was left up to siblings to support their “weaker” brothers and sisters:

“*Werner then 14 years old, was a problem. Never very strong, he had to be trained to carry a heavy suitcase” “Uncle Dieter and Aunt Ilse saw us off at the station in Bielefield. It was to be the last time I saw them. Tragically they and their little daughter were deported to the Warsaw Ghetto never to return*.”^32^

Accounts from former Kindertransport children and young people also highlight stigmas and labels in relation to their status, impacting on how they were received and treatment in the United Kingdom. For example, by having to report to the police:

*on the 10 th of December 1938 my parents took me to the Westbahnhof for my journey to Holland, originally, I was 14 yrs of age, this was the first Childrentransport from Vienna, of boys + girls of similar ages. Ironically we were classed as Enemy Aliens and had to report to Notting Hill Gate Police Station, as war broke out some of our boys were sent to Internment Camps in Canada*.^33^

Moreover, this also impacted the prospects and opportunities available to those young people after arrival in the United Kingdom:

*At the outset of the war, I was interned together with many other Jewish refugees, on the Isle of Man. I joined the British army in 1942 when I was 18 years old, having served the previous two years with the Home Guard in Bradford. As we were all considered enemy aliens in those early days of the war, I, together with many others, could only join the Pioneer Corps*.^34^

Here, as with the BHS, there is evidence that Kindertransport children/young people were positioned within a lower social class/hierarchy (Ala, 2018; Hopkins & Hill, 2010). In line with eugenicist perceptions of the time, a narrative of difference and deficiency was used to contain specific groups of people, including the poor (in case of the BHS) and groups from different ethnicities (in case of the Kindertransport scheme), ignoring and marginalizing their actual experiences (Kelly et al., 2021). In the case of the Kindertransport, this was also stimulated by the goal not to “replenish that good white stock with Jewish racial material” (Grenville, 2012, p. 4).

Here, it should be noted that in addition to being underpinned by biological determinism and eugenics, there were also practical reasons for why a child could not be migrated, especially in relation to the BHS, on long journeys to Canada and to a country much colder than the home country. An example of this is Thomas Patterson, who was not passed, because “cold could make eczema worse.”^35^ There was an inherent difficulty for the sick and disabled in migrating. There is a long history to this. On the one hand, imperial resettlement schemes prioritized the able bodied and healthy. On the other, in light of the Kindertransport, there were especial difficulties due to the Nazi persecution of the mentally and physically disabled. Provisions against infectious disease were extended to areas where there was no contagion but there were eugenic and racial concerns with corruption of the population (Grenville, 2012; Kelly et al., 2021).

## 5 DISCUSSION AND CONCLUSION

By critically reassessing and reflecting on the ideals and realities of two major historic child migration schemes, this paper adds to our current understanding of their place within wider international histories of child migration, moral reformism, eugenics, settlement, and identity. By reflecting on the varied experiences and narratives in relation to child migrants and the experiences of the children and young people “supported” through the schemes, we were able to provide insight into how both schemes were imbued with a eugenic ethos despite—or existing alongside—their ostensibly humanitarian aims. Both schemes have been described as “child rescue schemes,” and in contemporary debates the Kindertransport scheme is still used to reinforce Britain's image of “protector of vulnerable children” (Constantine, 2013). Yet, the Canadian (as well as more recently, the Australian) child migration schemes, although initially hailed as child rescue schemes, have been sporadically blighted by stories of child abuse and neglect. The most recent IICSA Inquiry and Report (IICSA, 2018) into the historical child migration schemes to Canada, Australia, New Zealand, and Southern Rhodesia highlights that child migration was never entirely uncontroversial: reports as far back as the 1800s expressed significant criticisms of it. Yet, politics and economic benefits were consistently prioritized over the welfare of children (Ala, 2018; Lynch, 2016; Parr, 1982).

This is no different for the Kindertransport scheme, which was motivated by political pragmatism blended with humanitarian compassion, largely ignoring the spiritual and emotional needs of the children (Kushner, 2012; Skelton, 1944). The image of the Kindertransport that has survived in British public memory (i.e. the notion that the Jewish child refugees were welcomed) is selective and flawed, and instead it could be argued that the Kindertransport was marked by marginalization, refused entry, and exclusion, with few exceptions (Kushner & Knox, 2012; McDonald, 2018). In essence, both the Kindertransport and British Home Child initiatives were permeated by paternalistic, classist and discriminatory attitudes. Moreover, in both schemes the ‘rescued' migrant child was generally seen as synonymous with “less worthy,” and children perceived as in poor health or mentally deficient were excluded (Sims‐Schouten et al., 2019; Weindling, 2020; Kushner & Knox, 2012; McDonald, 2018).

By viewing the realities of the child migrations schemes, including the varied experiences and narratives in relation to child migrants, in light of eugenicist narratives of difference, pathology, victimhood”, and contamination, we were able to shed a light on uneven practices, formations of power and expectations of the times (Kelly et al., 2021; Lynch, 2016; Moss et al., 2020). Although presented as moral programs to rescue children facing poverty (in case of the BHS schemes) or danger of traumatizing, racially degrading and ultimately genocidal persecution (in case of the Kindertransport scheme), there is evidence that only certain children “benefitted” from this, namely the physically and mentally healthy.

Research on historic child migration schemes has so far largely centered around the positive and negative experiences of the children/young people after arriving in the “promised land” (e.g., Constantine, 2002, 2013; Craig‐Norton, 2014). Phrased as “child rescue” schemes, the focus was (in the case of the current paper) on “rescuing” children, either from “inadequate” parents and surroundings (BHS) or the Nazi regime (Kindertransport) (Harris & Oppenheimer, 2001; Lynch, 2014). Yet, little research has shed a light on the children and young people who were left behind, excluded from the schemes, for mental or physical reasons. The current study is the first of its kind that utilizes a comparative analysis (comparing two historic child migration schemes) to provide insight into the decisions around which children and young people were of sufficient standard to be included in the schemes, highlighting the central role of eugenics and biological determinism (e.g., only “healthy” children could be included, as well as “good white stock” in relation to the BHS) and pragmatics (namely the fact that “healthy” children could contribute to society in the form of labor, and in essence pay their dues, rather than being a “drain” on society). Thus, what the above schemes have in common is that judgements regarding which children should be included/excluded in the schemes or returned to their country of origin (as was the case with children in the Canadian child migration scheme) were fueled by a type of eugenics oriented to transplanting strong physical and psychologically resilient specimens and discriminatory selection procedures (Sims‐Schouten et al., 2019; Weindling, 2020; Lynch, 2014). Opportunities to participate in the schemes (whether positive or negative) were limited to a select group, namely the mentally and physically able (Ala, 2018; Hopkins & Hill, 2010).

## CONFLICT OF INTERESTS

The authors declare that there are no conflict of interests.

## Data Availability

The data that support the findings of this study are available from the following libraries and archives: Children's Society Archive, London (formerly known as the Waifs and Strays Society); LAC (Library and Archives Canada), Ottawa; Jewish Community Archive, Vienna; Wiener Library, London. Restrictions apply to the availability of these data, which were used under license for this study. The data are available from the authors with the permission of the above named Library and Archives.

## Endnotes

1 LAC, W&S, A‐1139, 1911.

2 2 Jan., 27 Feb., and 10 March 1939, A/W 1971/2.

3 Central Archives for the History of the Jewish People in Jerusalem (http://cahjp.nli.org.il/).

4 LAC, W&S, 1929.

5 Wiener Library, 1368/2/2/117.

6 W&S, Case 2274, 1900.

7 LAC, W&S, 1918.

8 LAC, W&S, Chairman's Notes, 1910.

9 LAC, W&S, 1911.

10 LAC, W&S, Chairman's Report, nd.

11 LAC, W&S, Chairman's Notes, 1910.

12 LAC, W&S, Emigration Decisions File, 1911.

13 LAC, W&S Chairman's Notes, 1910.

14 LAC, Fegan, 1925.

15 LAC, Fegan, 1889.

16 LAC Fegan, 1922.

17 LAC, Fegan, 1925.

18 LAC, W&S, Memorandum, 1929.

19 LAC, W&S, Impressions of Toronto, 1929.

20 Hahn‐Warburg to Dr. Engel, 30 Dec. 1938, A/W 1970.

21 Exiner to IKG, 18 Jan. 1939, A/W 1971/2.

22 Exiner to IKG, 16 March 1939, A/W 1971/5.

23
http://www.uvm.edu/%7Elkaelber/children/

24 2 Jan., 27 Feb., and 10 March 1939, A/W 1971/2.

25 A/W 1971/17.

26 For Hans Lang see IKG records, microfilm 0884.0126.

27 Exiner to Schwarz, 22 Aug. 1939, A/W 1971/19.

28 IKG to RCM, A/W 1971/1.

29 IKG to RCM, 11 July 1939, ibid.

30
https://en.wikipedia.org/wiki/The_Reich_Family. http://www.europeana.eu/en/item/2059209/data_sounds_C0830X0080XX_0001

31
https://www.hansalbrechtfoundation.org/

32 Wiener Library, 1368/2/2/112.

33 Wiener Library, 1368/2/2/124.

34 Wiener Library, 1368/2/2/127.

35 LAC, W&S. Chairman's Notes.
